# Association between pre-pregnancy calcium intake and hypertensive disorders during the first pregnancy: the Japan environment and children’s study

**DOI:** 10.1186/s12884-020-03108-2

**Published:** 2020-07-28

**Authors:** Hyo Kyozuka, Tsuyoshi Murata, Toma Fukuda, Akiko Yamaguchi, Aya Kanno, Shun Yasuda, Akiko Sato, Yuka Ogata, Masahito Kuse, Mitsuaki Hosoya, Seiji Yasumura, Koichi Hashimoto, Hidekazu Nishigori, Keiya Fujimori, Japan Environment and Children’s Study (JECS) Group, Michihiro Kamijima, Shi Yamazaki, Yukihiro Ohya, Reiko Kishi, Nobuo Yaegashi, Koichi Hashimoto, Chisato Mori, Shuichi Ito, Zentaro Yamagata, Hidekuni Inadera, Takeo Nakayama, Hiroyasu Iso, Masayuki Shima, Youichi Kurozawa, Narufumi Suganuma, Koichi Kusuhara, Takahiko Katoh

**Affiliations:** 1 Fukushima Regional Center for the Japan Environmental and Children’s Study, 1 Hikarigaoka, 960-1295 Fukushima, Japan; 2 Department of Obstetrics and Gynecology, Fukushima Medical University School of Medicine, 1 Hikarigaoka, 960-1295 Fukushima, Japan; 3 Department of Pediatrics, Fukushima Medical University School of Medicine, 1 Hikarigaoka, 960-1295 Fukushima, Japan; 4 Department of Public Health, Fukushima Medical University School of Medicine, 1 Hikarigaoka, 960-1295 Fukushima, Japan; 5 Fukushima Medical Center for Children and Women, Fukushima Medical University, 1 Hikarigaoka, 960-1295 Fukushima, Japan

**Keywords:** Calcium intake, Preconception care, Hypertension, Hypertension disorder of pregnancy, Birth cohort study

## Abstract

**Background:**

Determining the appropriate preconception care to reduce the occurrence of hypertensive disorder of pregnancy (HDP) remains a challenge in modern obstetrics. This study aimed to examine the association between pre-pregnancy calcium (Ca) intake and HDP in normotensive primiparas.

**Methods:**

We used data from the Japan Environment Children’s study (JECS), which is the largest birth cohort study. A total of 33,894 normotensive Japanese primiparas were recruited for JECS between January 2011 and March 2014. Participants were categorized into five groups according to pre-pregnancy Ca intake quintiles (Q1 and Q5 were the lowest and highest Ca intake groups, respectively) to compare their basic background and obstetrics outcome. Multiple logistic regressions were performed to identify the effect of pre-pregnancy Ca intake on HDP, early onset HDP, and late-onset HDP, using Ca intake thresholds of 500, 550, 650, 700, 1000, 1500, and 1500 mg.

**Results:**

We found significant differences in maternal background among the Ca intake groups; in particular, there were more participants with low socioeconomic status, indicated by low education level and low household income, and smokers in the lowest Ca intake group. Multiple logistic regression did not show any significant difference with regard to HDP, early onset HDP, and late-onset HDP in each Ca intake threshold.

**Conclusions:**

Despite considerable recommendations concerning Ca intake for women of reproductive age, the present study indicates that pre-pregnancy Ca intake was not associated with an increased risk of new-onset hypertension among primiparas during pregnancy. Further studies examining the effect of other pre-pregnancy dietary factors on obstetric outcomes should be considered in the formulation of earlier preventive strategies for primiparas.

## Background

Hypertensive disorder of pregnancy (HDP) occurs in approximately 2.5% of all pregnancies in Japan [1]. This disorder is the direct cause in approximately 30,000 maternal deaths per year and accounts for 14% of maternal deaths worldwide [2, 3]. Pre-eclampsia (PE) and gestational hypertension (GH) are the main forms of HDP, conventionally defined as the new onset of hypertension after 20 weeks of gestation, with (PE) or without (GH) signs of organ dysfunction, including kidney, liver, and placenta. Because hypertension during pregnancy affects the long-term health of both the mother and offspring, a major challenge in modern obstetrics is to identify preventive strategies prior to pregnancy [4].

Calcium (Ca) is involved in many body functions [5]. Several reference values of daily Ca intake for reproductive age, which vary from 550 mg to 1300 mg, have been established [6–8]. Although studies have focused on the effect of Ca intake on bone health, few effects of Ca intake or supplements on other health outcomes have been have investigated. Blood pressure is regulated by intracellular calcium in vascular smooth muscle cell via vasoconstriction and variations of the vascular volume [9, 10]. Therefore, appropriate Ca intake for women who hope to conceive is essential not only for preventing future osteoporosis, which may be reduced by the combination of Ca with the appropriate 25-hydroxyvitamin D intake, but also for reducing the risk of HDP. Dietary factors have been suggested to play a role in the prevention of HDP, including GH and PE, and pre-pregnancy Ca intake is thought to an important factor to reduce the risk of HDP. In 1960, an epidemiological study reported a low prevalence of PE in Ethiopia, where the local diet contained a high amount of Ca [11]. Although several studies have examined the association between Ca intake and prevention of HDP, varying results have been reported, especially in the dose of Ca supplement. Some clinical studies have reported that a high dose of Ca supplementation (> 1.5 g/day) could prevent PE [12], while a systematic review reported that a low dose of Ca (< 1 g/day) may be sufficient (relative risk 0.38, 95% confidential interval (CI) 0.28–0.52) [13]. This evidence was used as the basis for the World Health Organization (WHO) recommendation for routine prenatal Ca supplementation of 1500–2000 mg daily beginning from the 20th gestational week for all pregnant women, particularly those residing in low-Ca intake areas whose populations are considered at a high risk [14].

Although enough evidence has shown a reduced risk of pregnancy-related hypertension by Ca supplementation during pregnancy, potential questions still remain whether pre-pregnancy Ca intake or low dose Ca supplementation will affect the risk of new-onset pregnancy-related hypertension disorder using a homogeneous large sample size.

Therefore, we aimed to examine whether pre-pregnancy maternal intake of Ca is associated with the occurrence of new-onset HDP, including PE and HDP among nullipara Japanese women, using data from a large cohort study.

## Methods

In this study, data from the Japan Environmental Children’s Study (JECS), a government-funded birth cohort study started in January 2011, were used. This survey investigated the effect of several environmental factors on children’s health [15]. Eligibility requirements of JECS participants (mothers) were as follows: (1) living in the study area at the time of application and were expected to live in Japan in the near future; (2) expected delivery date between August 1, 2011, and mid-2014; and (3) could participate without difficulty (i.e., they could answer the self-management questionnaire). Written informed consent was obtained from all participating women.

The JECS protocol was reviewed and approved by the Ministry of the Environment’s Institutional Review Board on Epidemiological Studies and by the Ethics Committees of all participating institutions. The JECS was conducted in accordance with the Helsinki Declaration and other nationally valid regulations and guidelines.

### Data collection

We used the dataset released in June 2016 (dataset: jecs-ag-20,160,424) for this study. This data set consisted of three types of information: (a) self-reported questionnaire obtained around the first trimester, including the maternal basic information, or food frequency questionnaires (FFQs); (b) self-reported questionnaire collected during their second/third trimester, including socioeconomic status such as maternal education or household income; and (c) obstetrics outcome and maternal medical background which was retrieved from the medical records of each subject’s institution.

In this study, we excluded cases with insufficient data, multiple pregnancies, multiparity, and hypertension before pregnancy.

### Determination of pre-pregnancy Ca intake, obstetric outcomes, and confounding factors

Ca intake before conception was determined using the FFQ, which was completed during the first trimester. Participants were asked how often they consumed various types of food from 1 year before pregnancy to their first trimester, which was taken to indicate dietary patterns during the preconception period. A standard portion size was specified for each item of the FFQ. Response options for the intake frequency ranged from almost never to ≥ 7 times/day for foods such as cheese, yogurt, and tofu and ranged from almost never to ≥ 10 glasses/day for beverages such as milk and fermented milk drinks. The intake frequencies then were multiplied by the specified portion size. Nutrient content of each food was obtained from the Japanese food consumption table 5th revised revision [16], and the daily intake of Ca was estimated by summing the contents from all the food items after multiplying by the frequency of consumption. This FFQ has been validated using 12-day weighed food records in adults aged 40–74 years [17]. This study considered only dietary Ca intake and did not include Ca supplementation.

Obstetric data included information with regard to HDP, gestational age at birth, and birth weight. HDP was defined as a new onset of hypertension (≥ 140/90 mmHg) after pregnancy. HDP was further classified into two categories: early onset (Eo) HDP (HDP occurred before 34 weeks) and late-onset (Lo) HDP (HDP occurred after 34 weeks). Preterm birth (PTB) was defined as delivery before 37 gestational weeks. Low birth weight (LBW) was defined as birth weight < 2500 g. The following items were used as confounding factors: advanced maternal age (AMA), pre-pregnancy body mass index (BMI), conception methods, maternal smoking status, maternal educational status, and annual household income. AMA at delivery was defined as maternal age ≥ 35 years. Participants were categorized into three BMI groups as follows: <18.5, 18.5–25.0, and ≥ 25.0 kg/m^2^ [1]. Conception methods were categorized into natural pregnancy or conception after some infertility treatment. Maternal participants were requested to provide information about their smoking status as follows: “never smoked,” “quit smoking before pregnancy,” “quit smoking during early pregnancy,” and “kept smoking during pregnancy.” “Kept smoking during pregnancy” was defined as the smoking category; otherwise, it was defined as non-smoking. Maternal education was categorized into four groups (junior high school, < 10; high school, 10–12; professional school or university, 13–16; and graduate school, ≥ 17 years). Annual household income was categorized into four levels (< 2,000,000; 2,000,000–5,999,999; 6,000,000–9,999,999; and ≥ 10,000,000 JPY) [1]. The inclusion criteria of confounding factors for this study were determined by clinical importance [18–21].

### Statistical analyses

First, participants were categorized according to quartile (Q1 as the lowest Ca intake group and Q5 as the highest Ca intake group) based on their daily Ca intake before pregnancy. Maternal characteristics and obstetric outcomes were summarized according to each group. Kruskal-Wallis (or One-way analysis of variance) test and chi-square tests were used to compare continuous and categorical variables, respectively. The cut off values for Ca intake (mg/day) for HDP were 500, 550, 650, 700, 1000, 1500, and 2000. Although some of these values have been established by WHO and others to account for health maintenance such as Ca needs for growth, development, and functioning, the thresholds were defined before the analysis based on literature [6, 8, 14]. For each Ca intake threshold, adjusted odds ratios and 95% CI for HDP, Eo-HDP, and Lo-HDP were calculated using a multiple logistic regression model, accounting for maternal age, conception method, pre-pregnancy BMI, maternal education, maternal smoking status, and household income. We accomplished this by using dummy variables for categorical variables composed of more than three categories. SPSS version 26 (IBM Corp., Armonk, NY) was used for the statistical analyses. A *p* value < 0.05 indicated statistical significance.

## Results

**Fig. 1:**
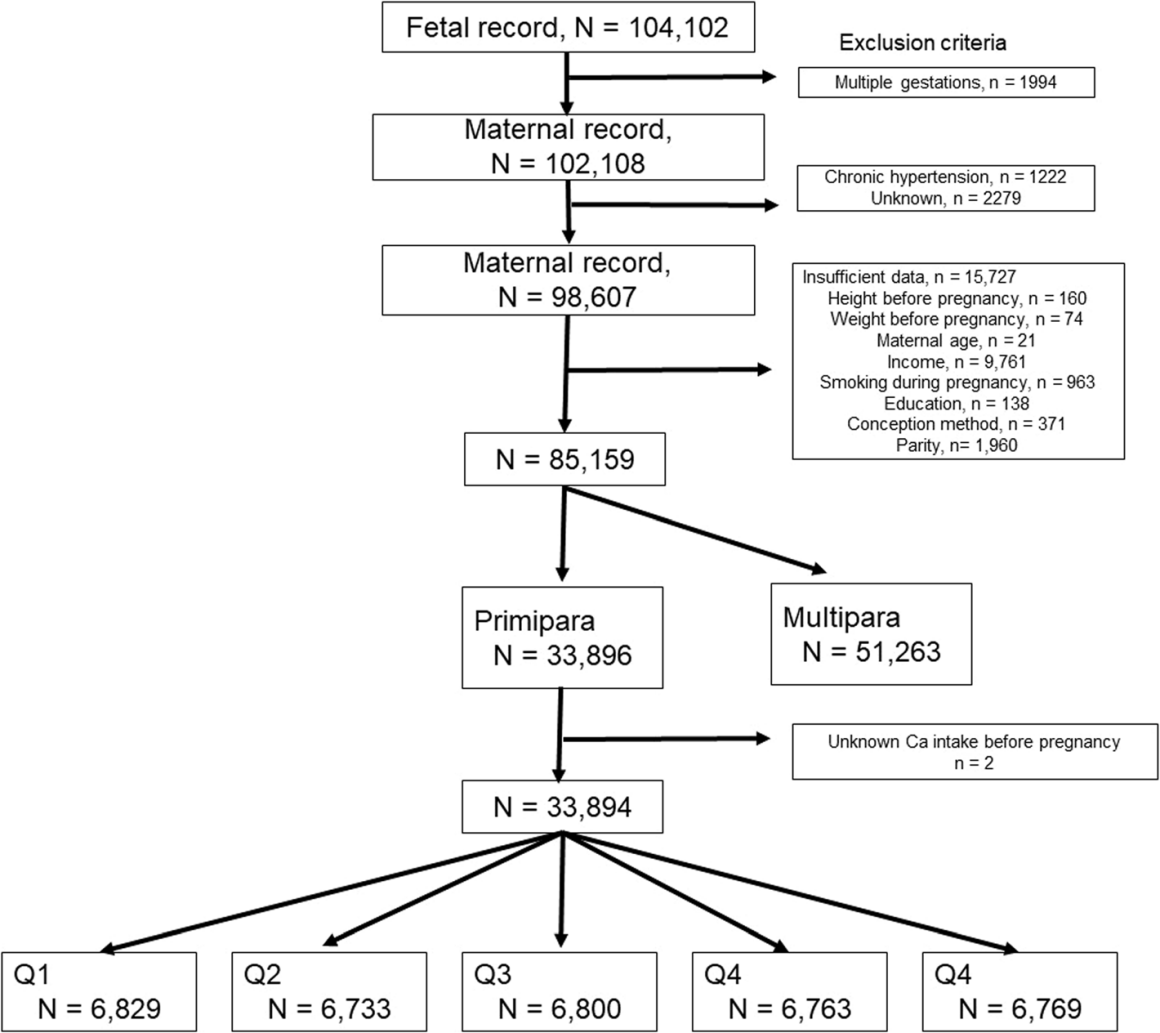
Study flow

In the JECS dataset, the total number of fetal records from infants delivered between 2011 and 2014 was 104,102. Of these, 1,994 infants from multiple gestations, 1,222 participants with maternal chronic hypertension, 15,727 participants with insufficient data, 51,263 multiparous participants, and two cases with unknown pre-pregnancy Ca intake were excluded. After applying our inclusion criteria, 33,894 participants were eligible for this study and categorized into five groups according to quintile (Fig. 1). The median (inter-quartile) pre-pregnancy Ca intake for participants was 430 (294–612) mg/day.

### Maternal medical and socioeconomic background and obstetric outcomes

Table 1 summarizes the maternal medical background and obstetric outcomes according to the quartile of pre-pregnancy Ca intake. The median (inter-quartile) pre-pregnancy Ca intake (mg/day) of each group from Q1 to Q5 was 199 (154–235), 321 (295–347), 430 (401–460), 567 (529–613), and 899 (755–1253) mg/day, respectively.

**Table 1:** Maternal medical background and obstetric outcomes per dietary Ca intake quintiles

Variable	Quintile for Ca					*p*-value
	Q1 (low)	Q2	Q3	Q4	Q5 (High)	
	*n* = 6829	*n* = 6733	*n* = 6800	*n* = 6763	*n* = 6769	
Maternal medical background						
Ca intake before pregnancy mg/day median (inter-quartile range)	199 (154–235)	321 (295–347)	430 (401–460)	567 (529–613)	899 (755–1253)	< 0.001^a^
Maternal age, years mean (SD)	28.5 (5.1)	29.9 (5.1)	30.4 (5.0)	30.7 (4.9)	30.8 (5.1)	< 0.001^b^
Advanced maternal age, %	13.4	19.2	21.3	22.9	24.2	< 0.001^c^
Pre-pregnancy BMI (kg/m^2^), %						
< 18.5	19.1	18.4	16.2	16.7	16.1	< 0.001^c^
18.5 to 25.0	71.4	72.4	75.3	75.0	76.2	
> 25.0	9.5	9.2	8.6	8.4	7.7	
Smoking during pregnancy, %	5.5	3.5	3.1	2.6	2.7	< 0.001^c^
Sterility treatment, %	7.3	10.7	10.8	11.5	12.2	< 0.001^c^
Maternal education, year, %						
< 10	5.8	3.6	2.6	2.3	2.5	< 0.001^c^
10 to 12	36.7	29.9	25.8	24.3	24.8	
13 to 16	39.6	43.0	43.2	43.2	43.9	
more than 17	17.7	23.6	28.5	30.2	28.8	
Household income, JPY, %						
<2,000,000	8.1	5.7	4.7	4.4	4.8	< 0.001^c^
2,000,000–5,999,999	69.5	66.4	65.6	64.1	64.1	
6,000,000–9,999,999	19.5	23.7	24.9	26.4	25.7	
≥10,000,000	3.0	4.2	4.9	5.1	5.3	
Obstetric outcome						
HDP, %	3.1	3.4	3.5	3.2	3.1	0.719^d^
Eo-HDP, %	0.5	0.7	0.5	0.5	0.6	0.739^d^
Lo-HDP, %	2.3	2.4	2.6	2.4	2.2	0.655^d^
PTB < 37 wks, %	4.4	3.9	4.5	4.2	4.0	0.322^d^
LBW < 2500 g, %	9.7	8.6	8.7	9.0	8.9	0.213^d^

*BMI* Body mass index, *Ca* Calcium, *Eo* Early onset, *HDP* Hypertensive disorder of pregnancy, *JPY* Japanese Yen, *LBW* Low birthweight, *Lo* Late onset, *PTB *Preterm birth, *SD *Standard deviation

^a^*p*-value, Kruskal-Wallis analysis

^b^*p*-value, one-way analysis of variance

^c^*p*-value, chi-square test

^d^*p*-value, the extended Mantel-Haenszel chi-square test

The mean maternal age was significantly different among the five groups (p < 0.001) and the highest in the Q5 group. The ratio of AMA and sterility treatment increased along with each category (p < 0.001 and p < 0.001, respectively). The rate of smoking, BMI > 25 kg/m^2^, maternal education < 10 years, and household income < 2.000.000 JPY, the lowest household income category, were significantly different among the five groups. All of these were the highest in Q1 group.

With regard to obstetric outcome, no significant differences were found in the occurrence of HDP (p = 0.719), Eo-HDP (p = 0.739), and Lo-HDP (p = 0.655).

### Pre-pregnancy Ca intake and risk of HDP

Table 2 summarizes the association between Ca intake and HDP, Eo-HDP, and Lo-HDP. The percentage of participants who had a dietary Ca intake greater than 500 mg/day, 550 mg/day, 650 mg/day, 700 mg/day, 1000 mg/day, 1500 mg/day, and 2000 mg/day were 38.9%, 32.1%, 21.6%, 18.0%, 7.9%, 3.3% and 2.0%, respectively. Pre-pregnancy calcium intake did not reduce the risk of HDP, Eo-HDP, and Lo-HDP at any threshold value.

**Table 2:** Relationship between Ca intake and hypertensive disorder of pregnancy

	Threshold of Ca intake						
	500 mg/day	550 mg/day	650 mg/day	700 mg/day	1000 mg/day	1500 mg/day	2000 mg/day
Participants with intake > threshold, %	38.9%	32.1%	21.6%	18.0%	7.9%	3.3%	2.0%
HDP							
Occurrence, %	3.3%	3.3%	3.3%	3.3%	3.2%	3.2%	3.2%
OR (95% CI)	0.96 (0.85–1.09)	0.98 (0.86–1.11)	0.95 (0.82–1.10)	0.90 (0.77–1.06)	1.04 (0.84–1.30)	1.25 (0.92–1.69)	1.25 (0.84–1.84)
aOR (95% CI)	0.96 (0.85–1.09)	0.98 (0.86–1.11)	0.95 (0.82–1.10)	0.89 (0.76–1.05)	1.04 (0.83–1.15)	1.20 (0.88–1.63)	1.22 (0.83–1.81)
Eo-HDP							
Occurrence, %	0.6%	0.6%	0.6%	0.6%	0.6%	0.6%	0.6%
OR (95% CI)	0.95 (0.71–1.27)	1.02 (0.75–1.38)	0.96 (0.68–1.36)	0.91 (0.63–1.34)	1.22 (0.75–1.99)	1.27 (0.63–2.59)	1.05 (0.39–2.84)
aOR (95% CI)	0.91 (0.68–1.23)	0.99 (0.73–1.34)	0.93 (0.65–1.32)	0.88 (0.60–1.29)	1.19 (0.73–1.95)	1.19 (0.58–2.43)	1.02 (0.38–2.77)
Lo-HDP							
Occurrence, %	2.4%	2.4%	2.4%	2.4%	2.4%	2.3%	2.4%
OR (95% CI)	0.97 (0.84–1.12)	0.97 (0.84–1.13)	0.94 (0.79–1.12)	0.88 (0.73–1.06)	1.04 (0.80–1.34)	1.26 (0.88–1.79)	1.20 (0.76–1.91)
aOR (95% CI)	0.97 (0.84–1.13)	0.97 (0.84–1.13)	0.94 (0.79–1.12)	0.88 (0.73–1.06)	1.03 (0.80–1.34)	1.22 (0.86–1.75)	1.19 (0.75–1.89)

*Ca* Calcium, *HDP* Hypertensive disorder of pregnancy, *Eo *Early onset, *Lo* Late onset, *OR* Odds ratio, *aOR* Adjusted odds ratio, *CI C*onfidence interval, *Ref* Reference

aOR was calculated by logistic regression analysis, using reference cut value (< threshold or more than threshold), maternal age (< 35 years), parity, method of conception, pre-pregnancy BMI, maternal smoking status, maternal education, and household income

## Discussion

To the best of our knowledge, this is the first study using a large prospective birth cohort in Japan to examine the effect of pre-pregnancy dietary Ca intake on the occurrence of new-onset hypertension during pregnancy among normotensive primiparas. Using data from the largest Japanese birth cohort study to date, normotensive primiparas were classified into five groups based on their daily dietary Ca intake from 1 year before pregnancy to their first trimester, because we expected to find associations between Ca intake and maternal background. Consequently, we found significant differences in maternal background among the Ca intake groups; in particular, there were more participants with low socioeconomic status (i.e., low education level and low household income) and current smoking status in the lowest Ca intake group. Recommendations for Ca intake in women of reproductive age have a wide range of values; however, in our analysis, when pre-pregnancy dietary Ca intake thresholds were set between 500 and 2000 mg/day, none of these threshold values showed a clear cutoff value to reduce the risk of HDP.

Compared with most previous studies, we targeted primiparas and focused on pre-pregnancy Ca intake, because of the limited evidence on the prevention of new-onset hypertension during pregnancy for this population. It is thought that subsequent pregnancies reduce the occurrence of PE because of higher implantation or placentation [22] and enhanced maternal cardiovascular adaptation [23–25] by increased end-diastolic blood volume, stroke volume, and decreased vascular resistance [24], resulting in decreased mean arterial pressure and reduced arterial stiffness [25]. Therefore, recurrent pregnancy itself has HDP preventive effects. Furthermore, women with a history of HDP are carefully managed and may be treated by low-dose aspirin, which has been well-established to prevent recurrent HDP [26]. In developed countries, the proportion of primiparas among the total number of deliveries is increasing [27], which may subsequently increase the number of women who have received insufficient prevention advice or treatment against new-onset hypertension. Therefore, one major challenge in modern obstetrics is formulating prevention strategies for HDP in primiparas. Another challenge is preconception care. Recently, interest in preconception health such as advantageous exercise, improved dietary habits, and smoking cessation is growing, as it is a crucial period for influencing not only pregnancy outcomes, but also future maternal and child health and prevention of chronic disorders. Women with a history of either GH or PE have a higher risk for diabetes mellitus and cardiovascular-related mortality and morbidity, including ischemic heart disease, stroke, and venous thromboembolism [28, 29]. With the viewpoint of preventing long-term sequelae, Berks et al. reported that lifestyle interventions for exercise, dietary habits, and smoking cessation decrease cardiovascular risk, with an odds ratio of 0.91 (95% CI 0.87–0.96) among women with a history of PE [30].

Although the pathogenesis of HDP has not been elucidated, several mechanisms for the biological effect of Ca intake during pregnancy on the risk of PE may exist. Low Ca intake increases calcitriol serum levels, stimulates parathyroid function, and renin secretion. The renin-angiotensin-aldosterone signaling pathway produces sodium and water resorption, resulting in increased vascular volume. Calcitriol may increase cytosolic free Ca concentration. The parathyroid gland secretes parathyroid hormone and the parathyroid factor. Both mediators increase cytosolic free Ca concentration, leading to the contraction of vascular smooth muscle tissue [31]. Therefore, Ca intake may affect smooth muscle function on utero-placental flow. Carroli et al. reported a trend toward lower pulsatility index and resistance index in uterine and umbilical arteries at every gestational stage among women who received Ca supplementation compared with women who received placebo [32].

The strength of this study is that it is the first large-scale study conducted in Japan with meticulous attention to data collection, including a large number of primiparas. This study is considered representative of the general pregnant population in Japan [33]. Although the present results were not derived from a randomized controlled study, the large-scale nature of this cohort study has advantages, allowing for evaluation of associations between obstetric outcomes and pre-pregnancy exposures.

Nevertheless, this study has several limitations. First, although we accounted for some confounding factors in large portions of the questionnaire, we did not consider daily Vitamin D intake. Vitamin D is a steroid hormone that can be synthesized in the skin under the influence of UV light or found in foods, such as fish and vegetables. Vitamin D deficiency stimulates the secretion of parathyroid hormone which, in turn, affects Ca concentration. Second, although obstetric outcomes were based on medical records, this study focused on HDP, which does not differentiate GH from PE. As PE is accompanied by proteinuria or other organ dysfunction, the complications of PE more severely affect the mother and infant than do those of GH [34]. Furthermore, we did not have information about the severity of HDP such as BP variations, urine protein determination, and organ dysfunction, which affect both short- and long-term maternal health conditions. Third, the FFQ, which was taken in the first trimester, includes information 1 year before from the pregnancy; therefore, recall bias may exist. Fourth, we did not evaluate of plasma Ca concentrations before pregnancy, which may be associated with Ca intake. Finally, we did not assess Ca intake during pregnancy, which may affect the occurrence of HDP.

## Conclusions

In conclusion, despite considerable recommendations concerning Ca intake for women of reproductive age, from the viewpoints of bone health and whether Ca intake during pregnancy could reduce the risk of new-onset HDP, we found no association between any pre-pregnancy Ca intake threshold and new-onset hypertension among primiparas during pregnancy. Because primiparas are vulnerable to new-onset HDP and daily diet could affect obstetric outcome, further studies that examine the effect of other pre-pregnancy dietary factors on obstetric outcomes should be considered in formulating earlier preventive strategies for primiparas.

## Abbreviations

AMA: Advanced maternal age
BMI: Body mass index
Ca: Calcium
CI: Confidence interval
Eo: Early onset
FFQs: Food frequency questionnaires
GH: Gestational hypertension
HDP: Hypertensive disorder of pregnancy
JECS: Japan Environment and Children’s Study
Lo: Late-onset
PE: Pre-eclampsia
RR: Relative risk
WHO: World Health Organization

## Availability of data and materials

The datasets analyzed during the current study are not publicly available due to confidentiality/research subject protections. The authors, with permission of the Eco-child Study Investigation Committee and the Japan government, can make the datasets available upon reasonable request.
